# Synergistic toxicity and physiological impact of imidacloprid alone and binary mixtures with seven representative pesticides on honey bee (*Apis mellifera*)

**DOI:** 10.1371/journal.pone.0176837

**Published:** 2017-05-03

**Authors:** Yu Cheng Zhu, Jianxiu Yao, John Adamczyk, Randall Luttrell

**Affiliations:** 1 USDA-ARS, Stoneville, Mississippi, United States of America; 2 USDA-ARS, Poplarville, Mississippi, United States of America; Institut Sophia Agrobiotech, FRANCE

## Abstract

Imidacloprid is the most widely used insecticide in the world. In this study, we used spraying methods to simulate field exposures of bees to formulated imidacloprid (Advise^®^ 2FL) alone and binary mixtures with seven pesticides from different classes. Synergistic toxicity was detected from mixtures of Advise (58.6 mg a.i./L imidacloprid)+Domark (512.5 mg a.i. /L tetraconazole), Advise+Transform (58.5 mg a.i./L sulfoxaflor), and Advise+Vydate (68 mg a.i./L oxamyl), and mortality was significantly increased by 20%, 15%, and 26% respectively. The mixtures of Advise+Bracket (88.3 mg a.i./L acephate) and Advise+Karate (62.2 mg a.i./L L-cyhalothrin) showed additive interaction, while Advise+Belay (9.4 mg a.i./L clothianidin) and Advise+Roundup (1217.5 mg a.i./L glyphosate) had no additive/synergistic interaction. Spraying bees with the mixture of all eight pesticides increased mortality to 100%, significantly higher than all other treatments. Except Bracket which significantly suppressed esterase and acetylcholinesterase (AChE) activities, other treatments of Advise-only and mixtures with other pesticides did not suppress enzyme activities significantly, including invertase, glutathione S-transferase (GST), and esterase and AChE. Immunity-related phenoloxidase (PO) activities in survivors tended to be more variable among treatments, but mostly still statistically similar to the control. By using specific enzyme inhibitors, we demonstrated that honey bees mainly rely on cytochrome P450 monooxygenases (P450s) for detoxifying Advise, while esterases and GSTs play substantially less roles in the detoxification. This study provided valuable information for guiding pesticide selection in premixing and tank mixing in order to alleviate toxicity risk to honey bees. Our findings indicated mixtures of Advise with detoxification-enzyme-inducing pesticides may help bees to detoxify Advise, while toxicity synergists may pose further risk to bees, such as the Bracket which not only suppressed esterase and AChE activities, but also increased toxicity to bees.

## Introduction

Honey bee (*Apis mellifera* Linnaeus) produces hundreds of millions of dollar worth of honey [1], and enhances crop value by approximately $12 billion through natural and commercialized pollination service annually in the United States [2–3]. However, honey bees are not immune to biological and physical threats. They are attacked by numerous pests, parasites, and pathogens [4–6]. In addition, honey bees are often adversely, although unintentionally, impacted by farming practices, resulting in losing favorable natural habitats and direct poisoning from pesticides, because honey bees utilize crops as forage and share the agroecosystem with other insects including the pests targeted by the pesticides.

With the widespread implementation of transgenic crops and concurrent decrease in the use of some pesticides, piercing/sucking insects have shifted from secondary pest status to serious pests [7–8]. This pest status shift, coupled with the development of insecticide resistance in target insects [9–10], has resulted in increased use of insecticides for seed treatments and foliar sprays of systemic insecticides. This also increased the risk of direct exposures of foraging bees to insecticides. Currently, a variety of insecticides are available for crop pest control, including pyrethroids, organophosphates, carbamates, and neonicotinoids. More than forty pesticides are currently recommended by extension specialists for the chemical control of row crop insects in US Midsouth area [11–13].

During the last decade, sublethal pesticide residues in pollen has become a major concern and possible contribution to honey bee colony decline. Neonicotinoids that are widely used for seed treatment [14] and foliar spray have been implicated as key insecticides in this issue. The possible relationships between honey bee colony losses and sublethal effects of pesticide residues have received considerable attention, and published data indicated that pesticide residues may pose a range of concerns from serious adverse impacts [15–23] to very low or no risk [24–26] to honey bees. While a significant research efforts have been placed on the impact of residue levels of pesticides on honeybees and the collective data from these studies are generally inconclusive, however, a number of important issues may have been ignored or received much less research attention. They include (1) many pesticides have both contact and systemic toxicities; (2) pesticide residues in pollen from one-time seed treatment might be significantly lower than the pesticide deposits on plant leaves and flowers from foliar sprays applied multiple times over a growing season; and (3) testing with technical grade (pure) chemical may ignore the synergistic toxicity from formulating reagents [27].

Imidacloprid was the first synthetic neonicotinoid insecticide commercialized in 1991, and it incurs toxicity through contact and oral ingestion. As same as other neonicotinoids, imidacloprid is an agonists of nicotinic acetylcholine receptor (nAChR). By acting on the central nervous system, neonicotinoids interfere with the transmission of stimuli by competing with the natural neurotransmitter acetylcholine. Irreversible and selective binding to the insect’s central nervous system causes paralysis and death by over-stimulation [28]. The systemic activity of imidacloprid is effective in controlling sucking insects, and the relatively low mammalian toxicity provide safety to users, thus making imidacloprid one of the most widely used insecticides [28].

Because sucking insects have become serious pests on southern row crops, especially cotton, in recent years, foliar sprays, almost an exclusive control method, are frequently applied [29] by growers using aerial sprayer or ground sprayer. Some crops with long blooming period are attractive to honey bees. While feeding method was widely used in previous toxicology studies to simulate in-hive feeding on contaminated pollens, the risks of foraging bees to direct and frequent exposures to foliar sprays (whole body exposure) and subsequently tarsal contact of pesticide residues on plant leaves have largely been less studied through laboratory simulation using spray tower. Further studies are needed to understand whether and how pesticide mixtures impact honey bee biology and physiology. In this study, we used a spray tower to simulate field spraying, instead using feeding or topical application (one-time exposure to limited body part). We also selected one of the imidacloprid commercial products, Advise^®^ 2FL (Advise) (used by farmers, instead of technical grade chemical) to examine potential additive/synergistic toxicities of Advise-only and binary mixtures (to simulate tank mixing, a common practice) with seven commonly used (representatives of different pesticide classes) pesticides to honey bee workers at LC_20_ concentration. In addition to mortality measurements, physiological responses were also measured using microtiter plate reader assays of several detoxification, metabolic-, and immune-related enzymatic activities in pesticide treated survivors. Furthermore, we explored detoxification mechanisms in honey bees through inhibition of detoxification enzymes.

## Methods and materials

### 2.1. Honey bee colony

Honey bee (Italian) queens and colonies were originally purchased from bee keepers located in pine forest and pasture area in Magee and Perkinston, Mississippi and maintained in a managed bee yard at the Mississippi Wildlife Management Area near Stoneville, MS. An oil trap (35x45 cm tray filled with vegetable oil) was installed at the bottom of each colony for *Varroa* mite (*Varroa destructor*) and small hive beetle (*Aethina tumida*) monitoring and control. Deep frames with more than 50% coverage of healthy sealed brood were pulled out and transferred to laboratory incubators (33°C±0.5°C; 65%±3 RH; no light). Twenty-five newly emerged workers were transferred daily into plastic cages (see descriptions below) and were provided a piece of global paddies (1 cm^3^, Betterbee, Greenwich, NY) placed at the bottom of the cage and one scintillation vial of 50% sugar solution and one scintillation vial of d-H_2_O at the top of the cage. Caged bees were maintained in incubators at the same conditions described above until being used for experiment.

### 2.2. Pesticides and test concentrations

Formulated imidacloprid Advise^®^ 2FL (Advise) and other insecticides were purchased from local agricultural chemical stores and kept in a refrigerator (6±1°C). Solutions of individual and mixture of pesticides, prepared by diluting chemical in d-H_2_O to a concentration equal to corresponding LC_20_ concentration for each pesticide, were used for spray treatments of Advise alone and mixtures with seven representative pesticides based on data of Zhu et al. [30]. These concentrations are: Advise at 274 mg/L; Bracket (acephate) at 91 mg/L; Karate (lambda-cyhalothrin) at 273 mg/L; Vydate (oxamyl) at 162 mg/L; Domark (tetraconazole) at 2500 mg/L; Roundup (glyphosate) at 2500 mg/L; Transform (sulfoxaflor) at 117 mg/L; Belay (clothianidin) at 40 mg/L. Details of pesticide name, manufacturer, percentage of active ingredient, spray concentration of formulation, and mode of action were listed in Table 1.

**Table 1:** Pesticide name, manufacturer, percentage of active ingredient, spray concentration of formulation, and mode of action.

Chemical name	Commercial name	Manufacturers	Active ingredient a.i.%	Spray concentration (at LC_20_)	LC_20_ a.i.	Mode of action
Imidacloprid	Advise 2FL	Winfield Solutions LLC	0.214	274	58.6	Nicotinic acetylcholine receptor (nAChR) competitive modulators [31]
Acephate	Bracket97	Winfield Solutions LLC	0.97	91	88.3	Acetylcholinesterase (AChE) inhibitors [31]
λ-Cyhalothrin	Karate Z 2.08 CS	Syngenta	0.228	273	62.2	Sodium channel modulators [31]
Oxamyl	Vydate 3.77 CLV	DuPont	0.42	162	68.0	Acetylcholinesterase (AChE) inhibitors [31]
Tetraconazole	Domark 230 ME	Valent	0.205	2500 *	512.5	Inhibit ergosterol biosynthesis enzyme C14-demethylase [32]
Glyphosate	Roundup PowerMAX	Monsanto	0.487	2500 **	1217.5	Glyphosate inhibits 5-enolpyruvylshikimic-3-phosphate synthase (EPSPS), causing a reduction of the biosynthesis of aromatic amino acids [33]
Sulfoxaflor	Transform 5G	Dow AgroSciences	0.5	117	58.5	Nicotinic acetylcholine receptor (nAChR) competitive modulators[31]
Clothianidin	Belay 50 WDG	Valent	0.236	40	9.4	Nicotinic acetylcholine receptor (nAChR) competitive modulators [31]

* The concentration used was 2.2-fold lower than LC_20_, which is 1.6-fold higher than recommended field use concentration.

** The concentration used was 1.849976E31-fold lower than LC_20_ and would be difficult to dilute if LC_20_ dose was used.

### 2.3. Bioassay methods

To simulate field foliar spray exposure, acute toxicities of Advise (imidacloprid) alone and binary mixtures with 7 representative pesticides were previously assayed using modified spray tower [30] for synergistic toxicity and specific enzyme inhibition tests. Briefly, the spray tower was constructed with Plexiglas^®^ to fit into a fume hood. The spray tower incorporated the original spray nozzle of Potter Spray Tower (Burkard Scientific Ltd, Uxbridge, UK) and nearly the same pressure air delivering and regulating systems as those in the Potter Spray Tower. Twenty-five newly emerged workers were transferred into a cage (made with a 500-ml round wide-mouth polypropylene jar [DxH: 9.3x10 cm]). An 8.9 cm diameter (d) hole was cut in the lid and covered with 3×3 mm-mesh metal screen for pesticide solution to spray through. Caged bees were supplied with 20 ml sugar syrupy (50%) and 20 ml d-H_2_O in separate scintillation vials and kept at 33°C for 8 days before being used for experiments. Three replicates (cages) were used for each treatment. Caged bees were sprayed once with 0.5 ml pesticide solution with air pressure at 69 kPa (10 psi) and spray distance of 22 cm. Sugar and water vials were removed during spraying and were placed back on the top of the cages after spraying. Sprayed (caged) bees were sent back to incubators (33°C) after spray treatments. Mortality was recorded 48-h after treatment. Three surviving bees were collected 48-h after treatment from each cage and were used for enzyme activity assays (below).

For specific enzyme inhibition test, caged bees were sprayed with 0.5 ml of PBO (piperonyl butoxide), TPP (triphenyl phosphate), or DEM (diethyl maleate) solution at 1% (in 50% acetone) one hour before spraying the Advise solution at 233 mg/L. Five replicates (cages) were included in each treatment.

### 2.4. Enzyme activity assays

#### 2.4.1. Chemicals

The following chemicals were purchased from Sigma-Aldrich (Sigma-Aldrich, St. Louis, MO): protease inhibitor (cocktail tablets), α-naphthyl acetate, fast blue B salt, 1-chloro-2,4-dinitrobenzene (CDNB), L-glutathione reduced (GSH), 4-nitrophenyl-α-D-glucopyranoside (pNPG), Dopamine hydrochloride, acetylthiocholine iodide (ATC), 5,5`-dithio-bis(2-nitrobenzoic acid) (DTNB), ρ-hydroxybenzhydrazide (PAHBAH), umbelliferone (7-hydroxycoumarin), 7-ethoxycoumarin (7-EC), oxidized glutathione (GSSG), glutathione reductase, β-nicotinamide adenine dinucleotide phosphate (reduced β-NADPH).

#### 2.4.2. Protein preparation

After 48 h of exposure to a single spray treatment, heads plus thoraxes of three surviving workers per replicate (3 replicates per treatment) from the treatment assay were ground in phosphate buffer pH7.2 with protease inhibitor and 0.3% triton X-100. The homogenization was centrifuged at 4°C, 20,800 × g, for 15min and the supernatant were collected for enzyme activity assays described below. Total protein concentration of each enzyme extraction sample was measured by Bradford protein assay kit [34] (ThermoScientific. Waltham, MA).

#### 2.4.3. Esterase activity assay

Esterase activity against α-naphthyl acetate was measured using the assay method of Zhu and Gao [35]. The homogenate was diluted by 5 fold with 0.1 M phosphate buffer pH7.5. The reaction solution consisted of 15 μl diluted enzyme and 135 μl 0.3 M α-naphthyl acetate. The reaction solution was incubated at 37°C for 30 min and the reaction was stopped by adding 50 μl fast Blue-SDS. Absorbance was recorded at 600 nm using a Synergy HTX plate reader (Bio-Tek Instruments Inc. Winooski, VT). The esterase activity was calculated based on a standard linear relationship established using α-naphthol.

#### 2.4.4. Glutathione S-transferase (GST) activity assay

GST activities were determined using the protocols of Yu [36] with some modifications. The reaction solutions (200 μl) contained 10 mM GSH, 2 mM CDNB, and 10 μl enzyme extraction. The optical density (OD) was continuously measured at 340 nm every 15 sec for a total of 10 min on Synergy HTX plate reader. The specific GST activity was calculated to nmol CDNB conjugation/min/mg protein using experimentally derived “extinction coefficients” of 5.3 mM^-1^cm^-1^ [37].

#### 2.4.5. Invertase activity assay

Invertase activity was determined using sucrose as substrate according to Lever [38] with some modification [39]. The reaction mixture of 100 μl enzyme extract and 900 μl 1% sucrose in 0.1 M pH4.5 acetate buffer were incubated at 55°C for 20 min in water bath. The reaction was stopped by mixing 50 μl reaction mixture with 1.45 ml 1% PAHBAH in 0.5 M sodium hydroxide solution and heated the mixture at 95°C for 5 min. The absorbance was measured at 410 nm. The activity of invertase was determined by hydrolyzing 1.0 μmole of sucrose to glucose and fructose per minute per mg protein at 55°C, pH 4.5.

#### 2.4.6. Phenoloxidase activity assay

The reaction solution contained 20 μl enzyme solution and 2 mM dopamine hydrochloride in pH 6.5 phosphate buffer. Phenoloxidase activity was measured at 490 nm for 30 min with 30-sec reading interval [40]. The activity of phenoloxidase was defined as the amount of enzyme which causes a change of OD 490 per minute per mg of protein in the reaction (units/min/mg protein).

#### 2.4.7. Acetylcholinesterase (AChE) activity assay

AChE activity was measured using acetylthiocholine (ATC) according to the method of Ellman et al. [41] with some modifications. Each reaction mixture included 50 μl enzyme extract, 0.25 mM ATC, and 0.4 mM DTNB in 150 μl of 0.1 M phosphate buffer pH7.5. The enzyme activity expressed by Vmax mOD/min was determined kinetically at 405 nm using Synergy HTX plate reader. AChE activities were expressed as nmol ATC hydrolyzed per min per mg protein using the extinction coefficient of 1.36×10^4^ M^−1^ cm^−1^.

### 2.5. Data processing and statistical analysis

SAS (version 9.2) [42] was used for analysis of variance (ANOVA). Proc GLM (general linear model) procedure was applied with option of Fisher’s LSD (least significant difference) method for mean separation at *P* = 0.05.

## Results

### 3.1 Synergistic/additive toxicity of Advise (imidacloprid) with other pesticides

**Fig 1:**
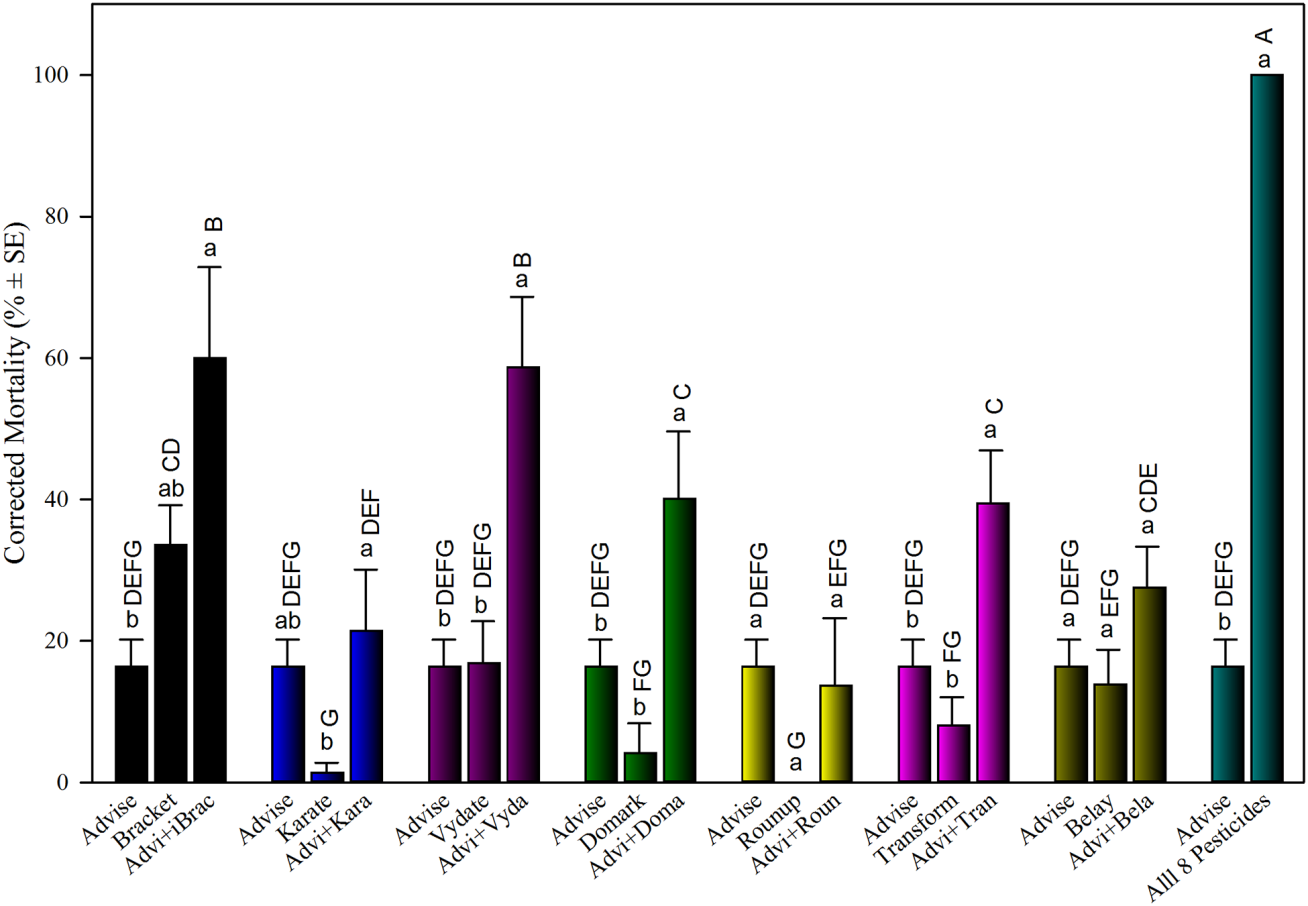
Acute toxicity of Advise (imidacloprid) and seven binary pesticide mixtures to honey bees. All concentrations used are listed in Table 1 as LC_20_ determined in a previous study [30]. Advi, Brac, Kara, Vyda, Doma, Roun, Tran, and Bela were used as abbreviations for the same order of eight pesticides listed in Table 1. Same letters above the error bars indicate no significant difference: lower case letters are for within-group comparisons; capital letters refer to comparisons among all treatments. The corrected mortality from Advise only was reused in each group for within-group comparison and statistics.

Toxicities of Advise were examined in binary mixtures with seven selected pesticides from different pesticide classes, all tested at LC_20_ levels except Domark and Roundup that were tested at 2,500 mg/L because they are not acutely toxic to honey bees [30]. Results showed that no bee died after 48 h in water-only control. Additive/synergistic toxicity was not detected from mixtures of Advise+Roundup and Advise+Belay (Fig 1). Synergistic toxicity was detected from binary mixtures of Advise+Domark, Advise+Transform, and Advise+Vydate, and mortality was significantly increased by 20%, 15%, and 26% compared to the added mortality of individual treatments, respectively. The mixtures of Advise+Bracket and Advise+Karate had additive toxicity with 10% and 4% higher mortality than the added of mortality of individual treatments, respectively. Roundup (glyphosate) at 2,500 mg/L did not kill bees, and the toxicity of the binary mixture of Advise+Roundup was not different from that of Advise-only treatment. The mixture of Advise with 7 other pesticides together killed 100% test bees, significantly higher than any individuals and binary mixtures and 6% higher than the added mortality of all 8 pesticides (Fig 1).

### 3.2 Effect of Advise and its binary mixtures on honey bee physiology

#### 3.2.1 Effects on insecticide-interacting enzymes: esterase (EST), glutathione S-transferase (GST), and acetylcholinesterase (AChE)

**Fig 2:**
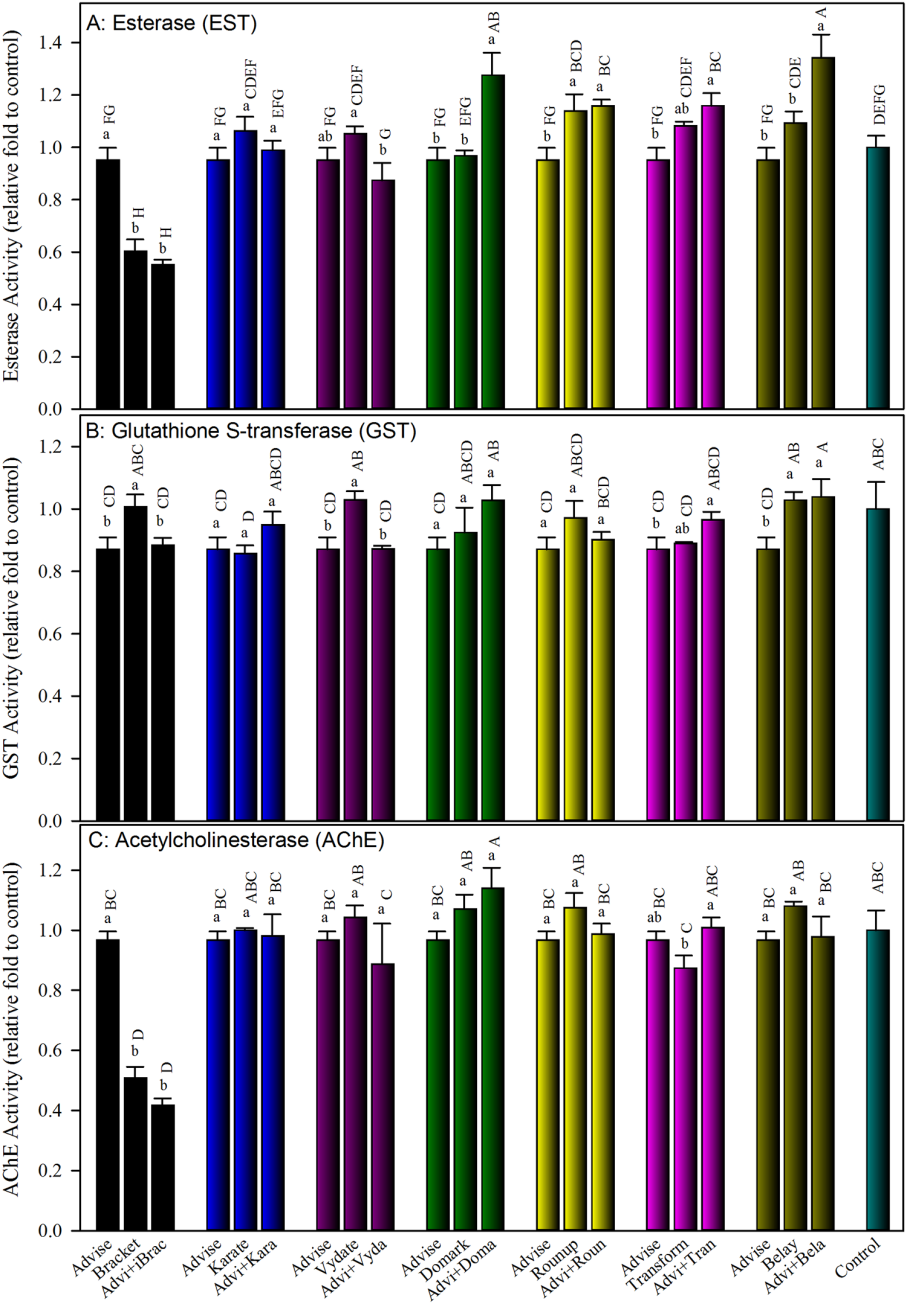
Impact of Advise and mixtures with seven representative pesticides on three insecticide-interacting enzymes, esterase (A), glutathione S-transferase (B), and acetylcholinesterase (C), activities in honey bee survivors after spray treatments. All concentrations used are listed in Table 1 as LC_20_ determined in a previous study [30]. Advi, Brac, Kara, Vyda, Doma, Roun, Tran, and Bela were used as abbreviations for the same order of eight pesticides listed in Table 1. Same letters above the error bars indicate no significant difference: lower case letters are for within-group comparisons; capital letters refer to comparisons among all treatments. The enzyme activity from Advise only was reused in each group for within-group comparison and statistics.

To make data comparable, enzymatic activities were converted to ratios of treatment to control. Results showed that most individual pesticide treatments and binary mixtures of Advise with six other pesticides had similar or higher esterase activities than untreated control, except for Bracket (acephate)-only and mixture of Advise+Bracket (Fig 2A). Bracket-only treatment reduced esterase activity by 40%, and the mixture of Advise+Bracket suppressed esterase activity by 45%. The mixture of Advise+Domark (fungicide) significantly induced 1.34- and 1.32-fold higher esterase activities than Advise- and Domark-only, respectively. Similarly, the mixture of Advise+Belay (clothianidin) significantly increased esterase activities by 1.41- and 1.23-fold than Advise- and Belay-only (Fig 2A).

Glutathione S-transferase (GST) activities were similar to that of the control in all cases except for the individual Karate (lambda-cyhalothrin) treatment that had significantly lower GST activity than control (Fig 2B). None of the mixtures had additive/synergistic or antagonistic effect on GST activity.

Acetylcholinesterase (AChE) activities were similar to that of the control in all cases except for both Bracket (organophosphate) treatments, individual and mixture with Advise, that had significantly lower AChE activity than control (Fig 2C). None of the binary mixtures of Advise with others produced significant additive/synergistic or antagonistic influence on AChE activity.

#### 3.2.2 Influences on invertase (INV: honey-making enzyme), and phenoloxidase (PO: immunity enzyme)

**Fig 3:**
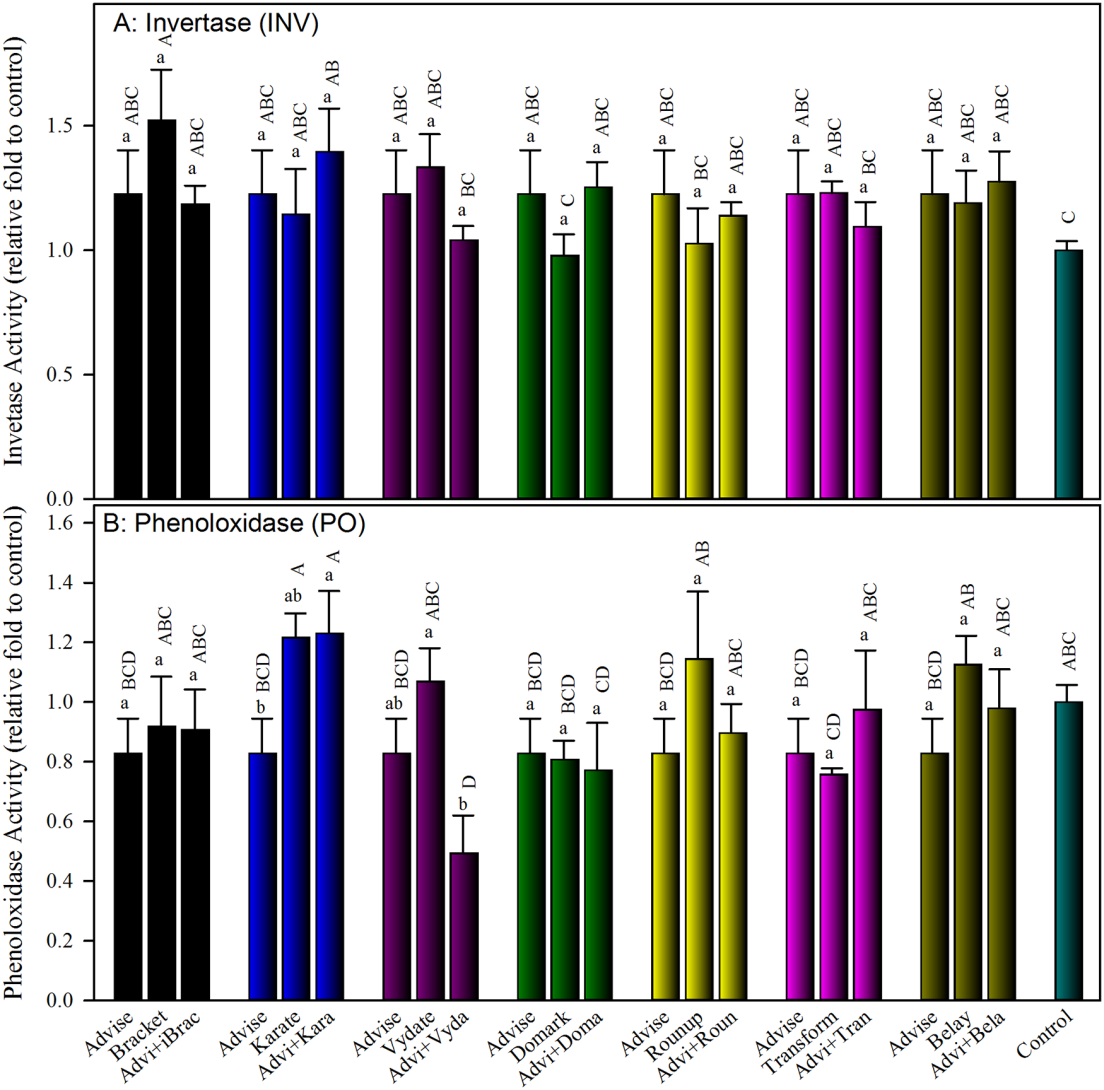
Impact of Advise and binary mixtures with seven representative pesticides on two non-pesticide-interacting enzyme, invertase (A) and phenoloxidase (B), activities in honey bee survivors after spray treatments. All concentrations used are listed in Table 1 as LC_20_ determined in a previous study [30]. Advi, Brac, Kara, Vyda, Doma, Roun, Tran, and Bela were used as abbreviations for the same order of eight pesticides listed in Table 1. Same letters above the error bars indicate no significant difference: lower case letters are for within-group comparisons; capital letters refer to comparisons among all treatments. The enzyme activity from Advise only was reused in each group for within-group comparison and statistics.

Most treatments of Advise and binary mixtures with 7 representative pesticides produced slightly higher INV activities, and no treatment produced significantly lower INV activities than control (Fig 3A). Two treatments, Bracket-only and Advise+Karate, produced significantly higher INV activities than control. Similarly, none of the binary mixtures of Advise with other pesticides induced significant additive/synergistic or antagonistic effect on INV activity.

Phenoloxidase activities in most treatments were slightly (but not significantly) lower than control, and only one treatment (Advise+Vydate) significantly reduced PO activities (Fig 3B). Slight (but not significant) increase of PO activities than control were seen only in 4 treatments, Advise+Karate, Karate, Roundup, and Belay. Similarly, none of the binary mixtures of Advise with others induced significant additive/synergistic or antagonistic impact on PO activity (Fig 3B).

### 3.3 Revealing of specific Advise-detoxification enzymes in honey bees using enzyme inhibitors

**Fig 4:**
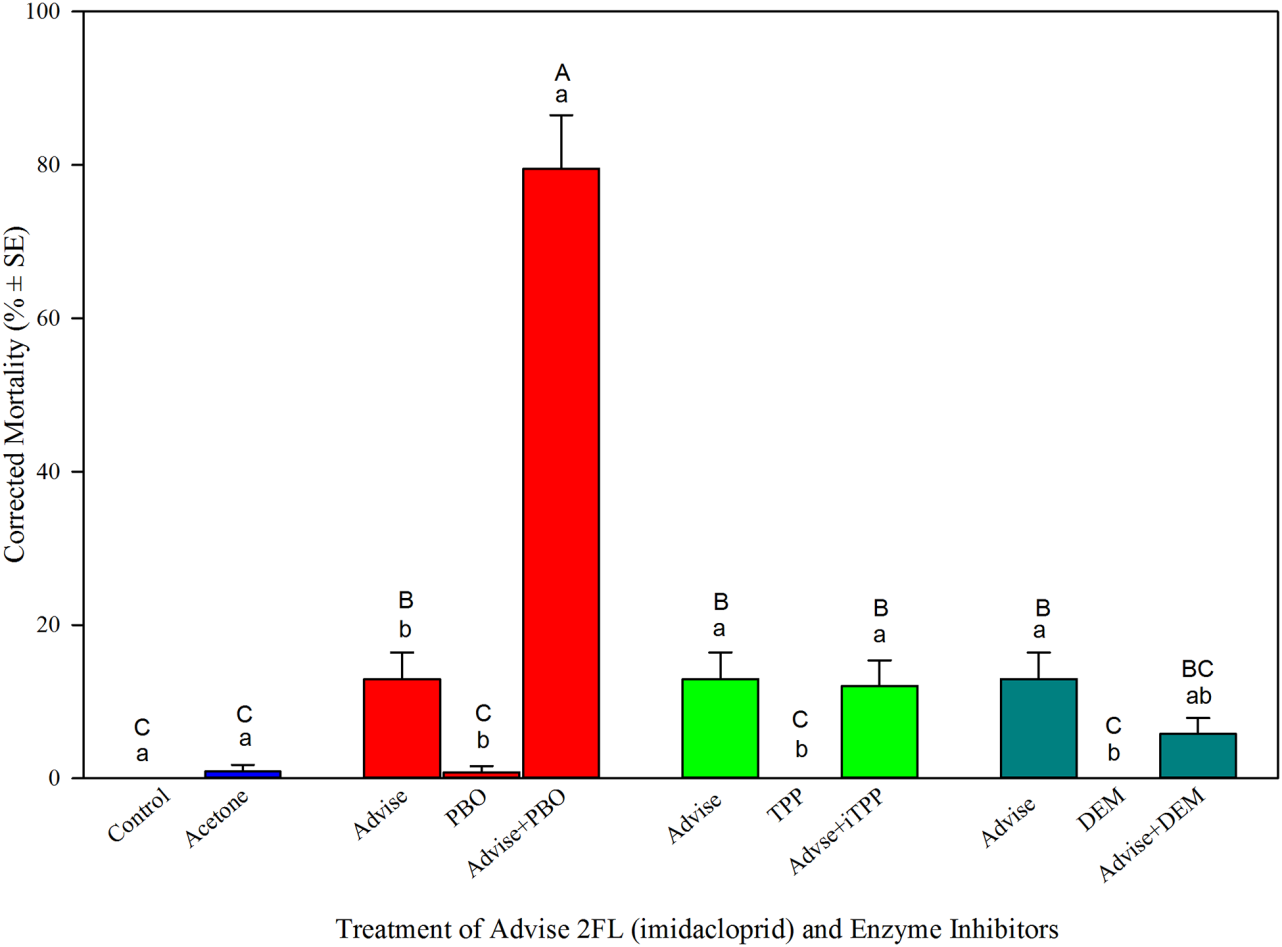
Roles of three major detoxification enzymes in detoxifying Advise (imidacloprid) in honey bees. PBO (piperonyl butoxide) is a cytochrome P450 oxidase inhibitor; TPP (triphenyl phosphate) is an esterase inhibitor; and DEM (diethyl maleate) is a glutathione S-transferase inhibitor. Same letters above the error bars indicate no significant difference: lower case letters are for within-group comparisons; capital letters refer to comparisons among all treatments.

Three specific enzyme inhibitors were applied to reveal the role of each enzyme in detoxifying Advise. Results (Fig 4) showed that PBO (piperonyl butoxide, P450 inhibitor”, synergistically enhanced Advise toxicity by 5.2-fold. TPP (triphenyl phosphate, esterase inhibitor) and DEM (diethyl maleate, GST inhibitor) did not synergize Advise’s toxicity against honey bees (Fig 4). The data indicated that P450 oxidases were involved in enhancing the toxicity of imidacloprid, while esterases and GSTs were not actively involved in Advise detoxification.

## Discussion

To achieve better control of multiple pests, farmers commonly mix formulated insecticides to simultaneously attack multiple targets [31,43]. The use of imidacloprid has been significantly increased since 2010 based on U.S. Geological Survey (https://water.usgs.gov/nawqa/pnsp/usage/maps/compound_listing.php). The increase may be related to the increases of treatments (seed treatment [44] and foliar spray [45]) and growing areas, and more than half of the imidacloprid was used on soybean and cotton. Up to 17 sprays were applied to cotton in 2015 in Mississippi Delta (http://www.entomology.msstate.edu/resources/cottoncrop.asp) for control of a variety of insects. Then honey bees may be exposed to mixtures of different pesticides in both in-hive and field situations. In this study, we focused on a neonicotinoid insecticide Advise, a formulation of imidacloprid which was one of the most concerning insecticides potentially related to honey bee declining and/or death [46–47]. To make binary mixtures with Advise, seven representative, also commonly used, pesticides for organophosphates, pyrethroids, carbamates, fungicides, herbicides, sulfoximines, and neonicotinoids were selected and applied to bees using spray tower to closely simulate field conditions. Subsequently, detecting additive/synergistic toxicities or interactions in some pesticide mixtures, revealing physiological responses to pesticide mixtures, and clarifying the importance of P450 monooxygenases in imidacloprid detoxification in this study contributed valuable information for guiding pesticide selections in order to reduce toxicity risk to bees by avoiding uses of toxicity synergizers.

Firstly, we provided a warning of potential toxicity increase to bees if certain pesticides are used in tank mixing with Advise (imidacloprid) after detections of significantly higher (synergistic) mortality than both individual pesticides from the mixtures of Advise with formulated Vydate (carbamate), Domark (fungicide), and Transform (sulfoximines). Besides these, Advise+Bracket (organophosphate) and Advise+Karate (pyrethroid) also produced higher mortality than either of the individual insecticides and this additive interaction need to be avoided during plant blooming stage. Advise+Roundup (herbicide) and +Belay (neonicotinoid) produced similar mortality to those produced by individual pesticides, therefore, these pesticides did not show any additive/synergistic interaction with Advise. However, detecting no significantly higher mortality in honey bees from these mixtures also demonstrated that pesticide mixtures or tank mixing are still feasible in synergizing toxicity and expanding control spectrum against crop pests without incurring further adverse impact on honey bees. All these interactions were obtained from mixtures prepared by mixing two individual chemicals both at LC_20_ concentrations. We do not exclude that these interactions may change if individual chemical concentration change or the ratio of two chemicals changes.

How other insecticides, fungicides, and herbicides interact with imidacloprid has not been well studied. Detection of potential synergistic toxicity at LC_20_ or higher concentration, while no detection of synergistic toxicity at lower (sublethal) concentration (data not shown), from the binary mixtures of Advise with other representative pesticides in this study indicated that individual chemical concentration have to reach certain threshold level to achieve intoxication together or facilitate the intoxication of the other party of the mixture, called “joint action” or “response addition” [48–49]. There are several commercialized formulated binary mixtures of neonicotinoids with pyrethroids, such as Endigo 2.06ZC (12.6% thiamethoxam +9.84% l-cyhalothrin) from Syngenta and Leverage 360EC (21% imidacloprid +10.5% b-cyfluthrin) from Bayer CropScience. These type of mixtures of two different insecticide classes may expand control spectrum to both lepidopterans and sucking insect pests. Pyrethroids produce excess nerve excitations by changing nerve membrane permeabilities to sodium and potassium ions, which is different from the competitive binding to nicotinic acetylcholine receptor by neonicotinoids [31]. Although we did not determine if the mixture of Advise with Karate (lambda-cyhalothrin) show any synergistic toxicity to crop pests, detecting no distinct synergistic toxicity to honey bees in this study would make this binary mixture more desirable for lessening risk to non-target pollinators.

Secondly, our *in vitro* examinations of activities of two detoxification enzymes (EST and GST), one insecticide-target enzyme (AChE), one metabolic enzyme (INV), and one immunity enzyme (PO) in surviving bees after treatments with Advise (imidacloprid) and mixtures added substantial knowledge on how pesticides impact honey bee physiology. Esterases (EST) are frequently implicated in the detoxification or resistance of insects to organophosphates, carbamates, and pyrethroids mainly through gene amplification and upregulation [50]. Glutathione S-transferase (GSTs) catalyze the secondary metabolism of a vast array of compounds oxidized by the cytochrome P450 family [51]. The catalytic reactions transform a wide range of endogenous and xenobiotic compounds, including herbicides and insecticides [52]. In this study, we detected no significant reduction of GST activities in survivors after bees were sprayed with LC_20_ concentrations of individual and binary mixtures of Advise with 7 representative pesticides (Fig 2). Except for the significant reduction of esterase activity by Bracket and Advise+Bracket, most esterase activities remained unchanged after being treated with individual and binary mixtures of Advise with other pesticides. Although most treatments of Advise and binary mixtures did not affect detoxification enzymes, our data also indicated that EST and GST played relatively less important roles in detoxifying neonicotinoids [53]. However, the roles of EST and GST in organophosphate toxicology cannot be ignored. Because it has both contact and systemic toxicities, Bracket (acephate) remains a preferred insecticide for tarnished plant bug control [10]. Thus, the decrease of detoxification enzyme activity (EST) by Bracket exposure in the field could weaken honeybee defense responses to other toxic chemicals, such as increased bee mortality in the treatment of Advise+Bracket in this study. Future studies are needed to understand how to reduce synergistic toxicity in honey bees by mixing with different insecticides or with different proportion of each pesticide in mixtures.

Acetylcholinesterase (AChE) inactivates the neurotransmitter acetylcholine in the synapses of the insect’s central nervous system [54–55]. In this study, we detected significantly lower AChE activity in Bracket-only- and Advise+Bracket-treated bees, and confirmed the inhibitory effects of organophosphate insecticides on AChE activity [55]. The AChE-suppression property, together with the EST-suppression and additive toxicity with Advise added further toxicity risk of Bracket to honey bees.

Invertase, the most important metabolic enzyme in honey, hydrolyzes nectar sucrose into fructose and glucose [56]. Our data indicated that invertase activity in survivors was not adversely influenced by Advise and mixtures with 7 representative pesticides at LC_20_ levels. It seemed that the eight pesticides tested in this study did not interfere with the sucrose-honey pathway (Fig 3A). The last enzyme we examined in this study was phenoloxidase, which is a key component of the insect immune system [57]. Overall, phenoloxidase activities in survivors were slightly (not significantly) reduced (Fig 3B), even though the same enzyme preparations were used for all five enzymes. The significant decrease of PO activities plus significant increase of mortality in Advise+Vydate-treated bees, necessitates a further investigations to understand any other factors that may affect PO regulation in treated bees and to understand how the interaction of Vydate with Advise influences PO activity pathway.

Finally, the confirmation of cytochrome P450 oxidases in honey bees as the major enzymes in detoxifying Advise (imidacloprid) established a mechanistic foundation for further investigations to sort out P450-inhibiting pesticides that need to be avoided in crop pest management program. We also confirmed that esterases (ESTs) and glutathione S-transferases (GSTs) played insignificant role in detoxifying Advise. However, we did not exclude the possible role of ESTs and GSTs in detoxification of other pesticides and the secondary metabolic compound of insecticides. The function of most P450 enzymes includes catalyzing the oxidation of organic substances to fulfill many important tasks, from the synthesis, degradation, and metabolic intermediations of lipids, ecdysteroids and juvenile hormones to the metabolism of xenobiotic substances of natural or synthetic origin [58]. P450 genes are also responsible in development of metabolic resistance to insecticides [59–60]. The inhibition on P450 may interfere these important biological and molecular processes in honey bees. However, how P450 genes are associated with imidacloprid detoxification has not been well established using P450 inhibitors, although P450 inhibitors effectively increased toxicity of cyano-group neonicotinoids to honey bees [53], but they did not effectively impact imidacloprid (nitro-group) [61]. It is possible that imidacloprid metabolites (5- hydroxyimidacloprid and olefin) have high affinity for the honey bee nAChR to induce bee mortality [61–62], and P450s may be still responsible for the production of these metabolites, although the metabolic process took longer than that for cyano-group neonicotinoids [53,55]. Nevertheless, any pesticide inducing P450s in honey bees and inhibiting P450s in target pests would be a desirable candidate for tank mixing or formulating premixtures.

Avoiding use of P450-inhibiting (to bees) pesticide in tank mixing is also an alternative and practical approach for reducing pesticide toxicity risk to bees. Domark (tetraconazole), belongs to the triazoles chemical group, is a broad spectrum fungicide that inhibits the metabolic pathway. Domark itself had relatively low spray toxicity to honey bees [30], and did not show distinct negative impact on five tested enzymes (Figs 2 and 3). However, we found Domark significantly synergized Advise toxicity to honey bees (Fig 1). The enhanced bee toxicity might be caused by the inhibition on cytochrome P450 enzyme from Domark [63]. Other studies [64–65] also reported that an imidazole fungicide, by inhibiting P450 enzymes, delayed the metabolism, detoxification, and excretion of λ-cyhalothrin, thereby effectively enhanced the toxicity of the pyrethroid to the honey bee. Our data provided a caution against the tank mixing of Domark with Advise and pyrethroid insecticides during plant blooming season due to its inhibitions to P450s, important detoxification enzymes in honey bees.

## Supporting information

S1 Data SupDataSpray8Pestc.pdf. (PDF)
