# Supplementary material for: Synergistic toxicity and physiological impact of imidacloprid alone and binary mixtures with seven representative pesticides on honey bee (Apis mellifera)
Source: PLoS One. 2017 May 3;12(5):e0176837. doi: 10.1371/journal.pone.0176837 (PMC5414993; doi:10.1371/journal.pone.0176837)
Supplement: S1 Data — (PDF) [file pone.0176837.s001.pdf]

Mortality data

input mixture $ treat $ rep dead0h total dead48h dead mortalit @;

cards;

CK-1	H2O	1	0	25	0	0	0.0000
CK-2	H2O	2	2	23	2	0	0.0000
CK-3	H2O	3	5	20	5	0	0.0000
AdvBra	Advi274B	1	2	23	5	3	0.1304
AdvBra	Advi274B	2	0	25	6	6	0.2400
AdvBra	Advi274B	3	0	25	3	3	0.1200
AdvBra	Brack91	1	3	22	10	7	0.3182
AdvBra	Brack91	2	0	25	11	11	0.4400
AdvBra	Brack91	3	1	24	7	6	0.2500
AdvBra	AdviBrack	1	0	25	20	20	0.8000
AdvBra	AdviBrack	2	0	25	16	16	0.6400
AdvBra	AdviBrack	3	0	25	9	9	0.3600
AdvKar	Advi274K	1	2	23	5	3	0.1304
AdvKar	Advi274K	2	0	25	6	6	0.2400
AdvKar	Advi274K	3	0	25	3	3	0.1200
AdvKar	Karat273	1	0	25	0	0	0.0000
AdvKar	Karat273	2	1	24	2	1	0.0417
AdvKar	Karat273	3	1	24	1	0	0.0000
AdvKar	AdviKara	1	1	24	2	1	0.0417
AdvKar	AdviKara	2	0	25	8	8	0.3200
AdvKar	AdviKara	3	0	25	7	7	0.2800
AdvVyd	Advi274V	1	2	23	5	3	0.1304
AdvVyd	Advi274V	2	0	25	6	6	0.2400
AdvVyd	Advi274V	3	0	25	3	3	0.1200
AdvVyd	Vydat162	1	4	21	10	6	0.2857
AdvVyd	Vydat162	2	1	24	4	3	0.1250
AdvVyd	Vydat162	3	4	21	6	2	0.0952
AdvVyd	AdviVyda	1	0	25	13	13	0.5200
AdvVyd	AdviVyda	2	1	24	12	11	0.4583
AdvVyd	AdviVyda	3	2	23	20	18	0.7826
AdvDom	Advi274D	1	2	23	5	3	0.1304
AdvDom	Advi274D	2	0	25	6	6	0.2400
AdvDom	Advi274D	3	0	25	3	3	0.1200
AdvDom	Doma2500	1	1	24	4	3	0.1250
AdvDom	Doma2500	2	2	23	2	0	0.0000
AdvDom	Doma2500	3	2	23	2	0	0.0000
AdvDom	AdviDoma	1	1	24	8	7	0.2917
AdvDom	AdviDoma	2	0	25	8	8	0.3200
AdvDom	AdviDoma	3	3	22	16	13	0.5909
AdvRou	Advi274R	1	2	23	5	3	0.1304
AdvRou	Advi274R	2	0	25	6	6	0.2400
AdvRou	Advi274R	3	0	25	3	3	0.1200
AdvRou	Roun2500	1	0	25	0	0	0.0000
AdvRou	Roun2500	2	0	25	0	0	0.0000

Enzyme data

input mix $ treat $ rep enzyme $ ActiFold @;

cards;

mix0	CK	1	GST	1.166826
mix0	CK	2	GST	0.957383
mix0	CK	3	GST	0.875792
mix0	2ck	1	GST	1.166826
mix0	2ck	2	GST	0.957383
mix0	2ck	3	GST	0.875792
mix0	3ck	1	GST	1.166826
mix0	3ck	2	GST	0.957383
mix0	3ck	3	GST	0.875792
mixb	AdviB	1	GST	0.836928
mixb	AdviB	2	GST	0.828455
mixb	AdviB	3	GST	0.946929
mixb	Brac	1	GST	0.931212
mixb	Brac	2	GST	1.025762
mixb	Brac	3	GST	1.063434
mixb	AdviBrac	1	GST	0.892903
mixb	AdviBrac	2	GST	0.837479
mixb	AdviBrac	3	GST	0.919398
mixk	AdviK	1	GST	0.836928
mixk	AdviK	2	GST	0.828455
mixk	AdviK	3	GST	0.946929
mixk	Kara	1	GST	0.827258
mixk	Kara	2	GST	0.909855
mixk	Kara	3	GST	0.833765
mixk	AdviKara	1	GST	0.98348
mixk	AdviKara	2	GST	0.865213
mixk	AdviKara	3	GST	0.999091
mixv	AdviV	1	GST	0.836928
mixv	AdviV	2	GST	0.828455
mixv	AdviV	3	GST	0.946929
mixv	Vyda	1	GST	1.0678
mixv	Vyda	2	GST	1.044419
mixv	Vyda	3	GST	0.975777
mixv	AdviVyda	1	GST	0.862423
mixv	AdviVyda	2	GST	0.860549
mixv	AdviVyda	3	GST	0.892603
mixd	AdviD	1	GST	0.836928
mixd	AdviD	2	GST	0.828455
mixd	AdviD	3	GST	0.946929
mixd	Doma	1	GST	0.780117
mixd	Doma	2	GST	0.938525
mixd	Doma	3	GST	1.055103
mixd	AdviDom	1	GST	1.124188
mixd	AdviDom	2	GST	0.991731

AdvRou	Roun2500	3	0	25	0	0	0.0000
AdvRou	AdviRoun	1	0	25	8	8	0.3200
AdvRou	AdviRoun	2	1	24	1	0	0.0000
AdvRou	AdviRoun	3	3	22	5	2	0.0909
AdvBel	Adv274BL	**1**	2	23	5	3	0.1304
AdvBel	Adv274BL	**2**	0	25	6	6	0.2400
AdvBel	Adv274BL	**3**	0	25	3	3	0.1200
AdvBel	Belay40	1	1	24	2	1	0.0417
AdvBel	Belay40	2	0	25	5	5	0.2000
AdvBel	Belay40	3	2	23	6	4	0.1739
AdvBel	AdviBela	1	1	24	9	8	0.3333
AdvBel	AdviBela	2	1	24	9	8	0.3333
AdvBel	AdviBela	3	0	25	4	4	0.1600
AdvTra	Advi274T	**1**	2	23	5	3	0.1304
AdvTra	Advi274T	**2**	0	25	6	6	0.2400
AdvTra	Advi274T	**3**	0	25	3	3	0.1200
AdvTra	Trans117	1	0	25	1	1	0.0400
AdvTra	Trans117	2	0	25	4	4	0.1600
AdvTra	Trans117	3	1	24	2	1	0.0417
AdvTra	AdviTran	1	2	23	14	12	0.5217
AdvTra	AdviTran	2	0	25	10	10	0.4000
AdvTra	AdviTran	3	2	23	8	6	0.2609
Adv8Pest	Advi2748	**1**	2	23	5	3	0.1304
Adv8Pest	Advi2748	**2**	0	25	6	6	0.2400
Adv8Pest	Advi2748	**3**	0	25	3	3	0.1200
Adv8Pest	8Pestici	1	0	25	25	25	1.0000
Adv8Pest	8Pestici	2	2	23	25	23	1.0000
Adv8Pest	8Pestici	3	0	25	25	25	1.0000
Adv8Pest	Pestic8	1	0	25	25	25	1.0000
Adv8Pest	Pestic8	2	2	23	25	23	1.0000
Adv8Pest	Pestic8	3	0	25	25	25	1.0000

```
run;
```

Specific inhibition data

input treat $ rep mixture $ mortalit @; cards;

control	1	CK	0
control	2	CK	0
control	3	CK	0
control	4	CK	0
control	5	CK	0
acetone	1	CK	4.35
acetone	2	CK	0
acetone	3	CK	0
acetone	4	CK	0
acetone	5	CK	0
AdviseP	1	PBO	4

mixd	AdviDom	3	GST	0.965243
mixr	AdviR	1	GST	0.836928
mixr	AdviR	2	GST	0.828455
mixr	AdviR	3	GST	0.946929
mixr	Roun	1	GST	1.078726
mixr	Roun	2	GST	0.902525
mixr	Roun	3	GST	0.931402
mixr	AdviRoun	1	GST	0.8559
mixr	AdviRoun	2	GST	0.940512
mixr	AdviRoun	3	GST	0.907553
mixbl	AdviBL	1	GST	0.836928
mixbl	AdviBL	2	GST	0.828455
mixbl	AdviBL	3	GST	0.946929
mixbl	Bela	1	GST	0.991218
mixbl	Bela	2	GST	1.014612
mixbl	Bela	3	GST	1.079115
mixbl	AdviBela	1	GST	0.957951
mixbl	AdviBela	2	GST	1.148515
mixbl	AdviBela	3	GST	1.00907
mixt	AdviT	1	GST	0.836928
mixt	AdviT	2	GST	0.828455
mixt	AdviT	3	GST	0.946929
mixt	Tran	1	GST	0.884647
mixt	Tran	2	GST	0.883413
mixt	Tran	3	GST	0.900054
mixt	AdviTran	1	GST	0.98443
mixt	AdviTran	2	GST	0.997147
mixt	AdviTran	3	GST	0.915281
mix0	CK	1	Est	1.04318
mix0	CK	2	Est	1.04472
mix0	CK	3	Est	0.9121
mix0	2ck	1	Est	1.04318
mix0	2ck	2	Est	1.04472
mix0	2ck	3	Est	0.9121
mix0	3ck	1	Est	1.04318
mix0	3ck	2	Est	1.04472
mix0	3ck	3	Est	0.9121
mixb	AdviB	1	Est	0.900738
mixb	AdviB	2	Est	0.908683
mixb	AdviB	3	Est	1.044496
mixb	Brac	1	Est	0.651975
mixb	Brac	2	Est	0.509723
mixb	Brac	3	Est	0.644935
mixb	AdviBrac	1	Est	0.578792
mixb	AdviBrac	2	Est	0.562499
mixb	AdviBrac	3	Est	0.515803
mixk	AdviK	1	Est	0.900738

AdviseP	2	PBO	24
AdviseP	3	PBO	8
AdviseP	4	PBO	12
AdviseP	5	PBO	16.7
PBO	1	PBO	0
PBO	2	PBO	0
PBO	3	PBO	0
PBO	4	PBO	0
PBO	5	PBO	4
AdviPBO	1	PBO	64
AdviPBO	2	PBO	81.6
AdviPBO	3	PBO	88
AdviPBO	4	PBO	100
AdviPBO	5	PBO	64
AdviseT	1	TPP	4
AdviseT	2	TPP	24
AdviseT	3	TPP	8
AdviseT	4	TPP	12
AdviseT	5	TPP	16.7
TPP	1	TPP	0
TPP	2	TPP	0
TPP	3	TPP	0
TPP	4	TPP	0
TPP	5	TPP	0
AdviTPP	1	TPP	8
AdviTPP	2	TPP	12
AdviTPP	3	TPP	24
AdviTPP	4	TPP	12
AdviTPP	5	TPP	4.17
AdviseD	1	DEM	4
AdviseD	2	DEM	24
AdviseD	3	DEM	8
AdviseD	4	DEM	12
AdviseD	5	DEM	16.7
DEM	1	DEM	0
DEM	2	DEM	0
DEM	3	DEM	0
DEM	4	DEM	0
DEM	5	DEM	0
AdviDEM	1	DEM	12
AdviDEM	2	DEM	4.17
AdviDEM	3	DEM	4
AdviDEM	4	DEM	0
AdviDEM	5	DEM	8.7
run;			

mixk	AdviK	2	Est	0.908683
mixk	AdviK	3	Est	1.044496
mixk	Kara	1	Est	1.158624
mixk	Kara	2	Est	0.970594
mixk	Kara	3	Est	1.059393
mixk	AdviKara	1	Est	1.006303
mixk	AdviKara	2	Est	0.918429
mixk	AdviKara	3	Est	1.042259
mixv	AdviV	1	Est	0.900738
mixv	AdviV	2	Est	0.908683
mixv	AdviV	3	Est	1.044496
mixv	Vyda	1	Est	1.039329
mixv	Vyda	2	Est	1.106371
mixv	Vyda	3	Est	1.009599
mixv	AdviVyda	1	Est	0.945079
mixv	AdviVyda	2	Est	0.7402
mixv	AdviVyda	3	Est	0.936306
mixd	AdviD	1	Est	0.900738
mixd	AdviD	2	Est	0.908683
mixd	AdviD	3	Est	1.044496
mixd	Doma	1	Est	0.944395
mixd	Doma	2	Est	1.00909
mixd	Doma	3	Est	0.950976
mixd	AdviDom	1	Est	1.282564
mixd	AdviDom	2	Est	1.419606
mixd	AdviDom	3	Est	1.124493
mixr	AdviR	1	Est	0.900738
mixr	AdviR	2	Est	0.908683
mixr	AdviR	3	Est	1.044496
mixr	Roun	1	Est	1.229198
mixr	Roun	2	Est	1.172149
mixr	Roun	3	Est	1.01334
mixr	AdviRoun	1	Est	1.109334
mixr	AdviRoun	2	Est	1.184475
mixr	AdviRoun	3	Est	1.18101
mixbl	AdviBL	1	Est	0.900738
mixbl	AdviBL	2	Est	0.908683
mixbl	AdviBL	3	Est	1.044496
mixbl	Bela	1	Est	1.136229
mixbl	Bela	2	Est	1.00292
mixbl	Bela	3	Est	1.138533
mixbl	AdviBela	1	Est	1.380168
mixbl	AdviBela	2	Est	1.473234
mixbl	AdviBela	3	Est	1.17191
mixt	AdviT	1	Est	0.900738
mixt	AdviT	2	Est	0.908683
mixt	AdviT	3	Est	1.044496

mixt	Tran	1	Est	1.102876
mixt	Tran	2	Est	1.093489
mixt	Tran	3	Est	1.049073
mixt	AdviTran	1	Est	1.073706
mixt	AdviTran	2	Est	1.242451
mixt	AdviTran	3	Est	1.159836
mix0	CK	1	AChE	1.104855
mix0	CK	2	AChE	1.01478
mix0	CK	3	AChE	0.880365
mix0	2ck	1	AChE	1.104855
mix0	2ck	2	AChE	1.01478
mix0	2ck	3	AChE	0.880365
mix0	3ck	1	AChE	1.104855
mix0	3ck	2	AChE	1.01478
mix0	3ck	3	AChE	0.880365
mixb	AdviB	1	AChE	0.922264
mixb	AdviB	2	AChE	0.955662
mixb	AdviB	3	AChE	1.022142
mixb	Brac	1	AChE	0.518259
mixb	Brac	2	AChE	0.438243
mixb	Brac	3	AChE	0.567404
mixb	AdviBrac	1	AChE	0.453453
mixb	AdviBrac	2	AChE	0.424103
mixb	AdviBrac	3	AChE	0.375142
mixk	AdviK	1	AChE	0.922264
mixk	AdviK	2	AChE	0.955662
mixk	AdviK	3	AChE	1.022142
mixk	Kara	1	AChE	0.999904
mixk	Kara	2	AChE	0.987559
mixk	Kara	3	AChE	1.012239
mixk	AdviKara	1	AChE	1.120917
mixk	AdviKara	2	AChE	0.882109
mixk	AdviKara	3	AChE	0.93918
mixv	AdviV	1	AChE	0.922264
mixv	AdviV	2	AChE	0.955662
mixv	AdviV	3	AChE	1.022142
mixv	Vyda	1	AChE	1.082095
mixv	Vyda	2	AChE	1.083049
mixv	Vyda	3	AChE	0.962919
mixv	AdviVyda	1	AChE	0.976266
mixv	AdviVyda	2	AChE	0.620053
mixv	AdviVyda	3	AChE	1.063125
mixd	AdviD	1	AChE	0.922264
mixd	AdviD	2	AChE	0.955662
mixd	AdviD	3	AChE	1.022142
mixd	Doma	1	AChE	0.975979
mixd	Doma	2	AChE	1.098239

mixd	Doma	3	AChE	1.135435
mixd	AdviDoma	1	AChE	1.222034
mixd	AdviDoma	2	AChE	1.192153
mixd	AdviDoma	3	AChE	1.004005
mixr	AdviR	1	AChE	0.922264
mixr	AdviR	2	AChE	0.955662
mixr	AdviR	3	AChE	1.022142
mixr	Roun	1	AChE	1.141109
mixr	Roun	2	AChE	1.103434
mixr	Roun	3	AChE	0.978342
mixr	AdviRoun	1	AChE	0.918632
mixr	AdviRoun	2	AChE	1.037766
mixr	AdviRoun	3	AChE	1.003027
mixbl	AdviBL	1	AChE	0.922264
mixbl	AdviBL	2	AChE	0.955662
mixbl	AdviBL	3	AChE	1.022142
mixbl	Bela	1	AChE	1.057557
mixbl	Bela	2	AChE	1.072632
mixbl	Bela	3	AChE	1.109905
mixbl	AdviBela	1	AChE	0.965369
mixbl	AdviBela	2	AChE	1.101466
mixbl	AdviBela	3	AChE	0.86446
mixt	AdviT	1	AChE	0.922264
mixt	AdviT	2	AChE	0.955662
mixt	AdviT	3	AChE	1.022142
mixt	Tran	1	AChE	0.830135
mixt	Tran	2	AChE	0.956967
mixt	Tran	3	AChE	0.834796
mixt	AdviTran	1	AChE	0.979376
mixt	AdviTran	2	AChE	1.076134
mixt	AdviTran	3	AChE	0.969853
mix0	CK	1	PO	1.035769
mix0	CK	2	PO	0.887642
mix0	CK	3	PO	1.076589
mix0	2ck	1	PO	1.035769
mix0	2ck	2	PO	0.887642
mix0	2ck	3	PO	1.076589
mix0	3ck	1	PO	1.035769
mix0	3ck	2	PO	0.887642
mix0	3ck	3	PO	1.076589
mixb	AdviB	1	PO	1.037107
mixb	AdviB	2	PO	0.633852
mixb	AdviB	3	PO	0.813969
mixb	Brac	1	PO	0.715258
mixb	Brac	2	PO	1.249049
mixb	Brac	3	PO	0.793034
mixb	AdviBrac	1	PO	1.151625

mixb	AdviBrac	2	PO	0.88436
mixb	AdviBrac	3	PO	0.685348
mixk	AdviK	1	PO	1.037107
mixk	AdviK	2	PO	0.633852
mixk	AdviK	3	PO	0.813969
mixk	Kara	1	PO	1.210676
mixk	Kara	2	PO	1.358588
mixk	Kara	3	PO	1.081273
mixk	AdviKara	1	PO	1.49986
mixk	AdviKara	2	PO	1.019844
mixk	AdviKara	3	PO	1.171686
mixv	AdviV	1	PO	1.037107
mixv	AdviV	2	PO	0.633852
mixv	AdviV	3	PO	0.813969
mixv	Vyda	1	PO	1.256939
mixv	Vyda	2	PO	0.872083
mixv	Vyda	3	PO	1.079628
mixv	AdviVyda	1	PO	0.746742
mixv	AdviVyda	2	PO	0.365168
mixv	AdviVyda	3	PO	0.368815
mixd	AdviD	1	PO	1.037107
mixd	AdviD	2	PO	0.633852
mixd	AdviD	3	PO	0.813969
mixd	Doma	1	PO	0.829428
mixd	Doma	2	PO	0.903781
mixd	Doma	3	PO	0.691093
mixd	AdviDom	1	PO	0.756975
mixd	AdviDom	2	PO	0.504047
mixd	AdviDom	3	PO	1.053665
mixr	AdviR	1	PO	1.037107
mixr	AdviR	2	PO	0.633852
mixr	AdviR	3	PO	0.813969
mixr	Roun	1	PO	0.839719
mixr	Roun	2	PO	1.008924
mixr	Roun	3	PO	1.585287
mixr	AdviRoun	1	PO	0.7068
mixr	AdviRoun	2	PO	1.021506
mixr	AdviRoun	3	PO	0.962446
mixbl	AdviBL	1	PO	1.037107
mixbl	AdviBL	2	PO	0.633852
mixbl	AdviBL	3	PO	0.813969
mixbl	Bela	1	PO	0.958831
mixbl	Bela	2	PO	1.292614
mixbl	Bela	3	PO	1.124852
mixbl	AdviBela	1	PO	0.758043
mixbl	AdviBela	2	PO	1.209121
mixbl	AdviBela	3	PO	0.970108

mixt	AdviT	1	PO	1.037107
mixt	AdviT	2	PO	0.633852
mixt	AdviT	3	PO	0.813969
mixt	Tran	1	PO	0.73229
mixt	Tran	2	PO	0.739077
mixt	Tran	3	PO	0.799622
mixt	AdviTran	1	PO	1.35822
mixt	AdviTran	2	PO	0.69619
mixt	AdviTran	3	PO	0.870368
mix0	CK	1	INV	0.960366
mix0	CK	2	INV	0.965512
mix0	CK	3	INV	1.074122
mix0	2ck	1	INV	0.960366
mix0	2ck	2	INV	0.965512
mix0	2ck	3	INV	1.074122
mix0	3ck	1	INV	0.960366
mix0	3ck	2	INV	0.965512
mix0	3ck	3	INV	1.074122
mixb	AdviB	1	INV	0.875327
mixb	AdviB	2	INV	1.369971
mixb	AdviB	3	INV	1.432082
mixb	Brac	1	INV	1.851257
mixb	Brac	2	INV	1.563688
mixb	Brac	3	INV	1.148929
mixb	AdviBrac	1	INV	1.243135
mixb	AdviBrac	2	INV	1.038341
mixb	AdviBrac	3	INV	1.275285
mixk	AdviK	1	INV	0.875327
mixk	AdviK	2	INV	1.369971
mixk	AdviK	3	INV	1.432082
mixk	Kara	1	INV	0.779818
mixk	Kara	2	INV	1.327239
mixk	Kara	3	INV	1.326776
mixk	AdviKara	1	INV	1.665061
mixk	AdviKara	2	INV	1.071374
mixk	AdviKara	3	INV	1.452618
mixv	AdviV	1	INV	0.875327
mixv	AdviV	2	INV	1.369971
mixv	AdviV	3	INV	1.432082
mixv	Vyda	1	INV	1.0715
mixv	Vyda	2	INV	1.460921
mixv	Vyda	3	INV	1.471176
mixv	AdviVyda	1	INV	0.931914
mixv	AdviVyda	2	INV	1.124848
mixv	AdviVyda	3	INV	1.064632
mixd	AdviD	1	INV	0.875327
mixd	AdviD	2	INV	1.369971

mixd	AviD	3	INV	1.432082
mixd	Doma	1	INV	0.908655
mixd	Doma	2	INV	0.878113
mixd	Doma	3	INV	1.149408
mixd	AdviDoma	1	INV	1.356537
mixd	AdviDoma	2	INV	1.051919
mixd	AdviDoma	3	INV	1.352098
mixr	AdviR	1	INV	0.875327
mixr	AdviR	2	INV	1.369971
mixr	AdviR	3	INV	1.432082
mixr	Roun	1	INV	1.227438
mixr	Roun	2	INV	1.101355
mixr	Roun	3	INV	0.754275
mixr	AdviRoun	1	INV	1.032561
mixr	AdviRoun	2	INV	1.205157
mixr	AdviRoun	3	INV	1.17944
mixbl	AdviBL	1	INV	0.875327
mixbl	AdviBL	2	INV	1.369971
mixbl	AdviBL	3	INV	1.432082
mixbl	Bela	1	INV	1.059717
mixbl	Bela	2	INV	1.451362
mixbl	Bela	3	INV	1.058425
mixbl	AdviBela	1	INV	1.066759
mixbl	AdviBela	2	INV	1.272521
mixbl	AdviBela	3	INV	1.48816
mixt	AdviT	1	INV	0.875327
mixt	AdviT	2	INV	1.369971
mixt	AdviT	3	INV	1.432082
mixt	Tran	1	INV	1.140373
mixt	Tran	2	INV	1.300494
mixt	Tran	3	INV	1.249976
mixt	AdviTran	1	INV	1.230278
mixt	AdviTran	2	INV	1.151975
mixt	AdviTran	3	INV	0.903975

run;
